# *Bacillus aryabhattai* SRB02 tolerates oxidative and nitrosative stress and promotes the growth of soybean by modulating the production of phytohormones

**DOI:** 10.1371/journal.pone.0173203

**Published:** 2017-03-10

**Authors:** Yeon-Gyeong Park, Bong-Gyu Mun, Sang-Mo Kang, Adil Hussain, Raheem Shahzad, Chang-Woo Seo, Ah-Yeong Kim, Sang-Uk Lee, Kyeong Yeol Oh, Dong Yeol Lee, In-Jung Lee, Byung-Wook Yun

**Affiliations:** 1 School of Applied Biosciences, Kyungpook National University, Daegu, Republic of Korea; 2 Department of Agriculture, Abdul Wali Khan University, Mardan, Khyber Pakhtunkhwa, Pakistan; 3 Gyeongnam Oriental Medicinal Herb Institute, Sancheong, Republic of Korea; Estacion Experimental del Zaidin, SPAIN

## Abstract

Plant growth promoting rhizobacteria (PGPR) are diverse, naturally occurring bacteria that establish a close association with plant roots and promote the growth and immunity of plants. Established mechanisms involved in PGPR-mediated plant growth promotion include regulation of phytohormones, improved nutrient availability, and antagonistic effects on plant pathogens. In this study, we isolated a bacterium from the rhizospheric soil of a soybean field in Chungcheong buk-do, South Korea. Using 16S rRNA sequencing, the bacterium was identified as *Bacillus aryabhattai* strain SRB02. Here we show that this strain significantly promotes the growth of soybean. Gas chromatography—mass spectrometry analysis showed that SRB02 produced significant amounts of abscisic acid, indole acetic acid, cytokinin and different gibberellic acids in culture. SRB02-treated soybean plants showed significantly better heat stress tolerance than did untreated plants. These plants also produced consistent levels of ABA under heat stress and exhibited ABA-mediated stomatal closure. High levels of IAA, JA, GA12, GA4, and GA7, were recorded in SRB02-treated plants. These plants produced longer roots and shoots than those of control plants. *B*. *aryabhattai* SRB02 was found to be highly tolerant to oxidative stress induced by H_2_O_2_ and MV potentiated by high catalase (CAT) and superoxide dismutase (SOD) activities. SRB02 also tolerated high nitrosative stress induced by the nitric oxide donors GSNO and CysNO. Because of these attributes, *B*. *aryabhattai* SRB02 may prove to be a valuable resource for incorporation in biofertilizers and other soil amendments that seek to improve crop productivity.

## Introduction

In natural ecosystems, plants interact with other organisms that make up the bulk of soil flora and fauna. These interactions determine the microbial diversity of the soil, although not all of these interactions are beneficial for the plants. Rhizobacteria that are found in the root zone (rhizosphere) of plants colonize and interact with plant roots. Although parasitic species of rhizobacteria exist, the term “rhizobacteria” is ordinarily used only in the context of mutually beneficial or symbiotic associations in which the bacteria help in the promotion of plant growth. That is why these bacteria are also called plant growth promoting rhizobacteria (PGPR) [1, 2]. Plant growth promotion may come about through direct regulation of phytohormonal activity, increasing root surface area, increasing tolerance to plant diseases [3–5], rhizosphere engineering, siderophore production, phosphate solubilization, and the production of active chemical signals [6, 7]. These phenomena have been observed in plant-PGPR interactions involving agriculturally important crops such as canola [8], pepper [9], tomato [10], cereals and legumes [11], and forest trees [12, 13]. PGPR have also been used for phytoremediation of contaminated soils [14]. Their growth promoting effects have been observed during a variety of stress conditions such as salinity [2, 15], drought [16, 17], heat stress [18], metal toxicity [19], and others. For these reasons, PGPR are now widely used as biofertilizers, soil amendments, and rhizoremediators [20–22]. Although the positive effects of PGPR on plant growth have been well known for decades, the underlying molecular mechanisms of plant growth promotion are poorly understood [23].

*Pseudomonas aeruginosa*, when inoculated to soil with Zn toxicity, improved the growth of wheat plants and their tolerance to oxidative stress as shown by an increase in the activity of antioxidant enzymes such as superoxide dismutase (SOD), peroxidase (POD), and catalase (CAT) [19]. Pepper plants inoculated with *Bacillus licheniformis* strain K11 tolerated drought stress more efficiently than un-inoculated plants, which died after 15 days of drought treatment [17]. Another study involving five different species of bacteria—*Bacillus subtilis* EY2, *Bacillus atrophaeus* EY6, *Bacillus spharicus* EY30, *Staphylococcus kloosii* EY37, and *Kocuria erythromyxa* EY43 found that these PGPR, were able to mitigate the deleterious effects of salt stress in strawberry plants [15]. Heat stress has been a limiting factor to agricultural production throughout the world, especially during dry periods and in areas on and near the equator. In such areas, the interactions of plants with different PGPR play important roles in ameliorating heat stress. For example, heat stress tolerance of wheat plants was increased after treatment with *Pseudomonas aeruginosa* 2CpS1 [18], *Bacillus amyloliquefaciens* UCMB5110 and *Azospirillum brasilense* NO40 [24], and *Pseudomonas putida* AKMP7 [25]. However, the molecular mechanisms underpinning heat stress tolerance of plants after PGPR treatment are largely unknown.

PGPR have been shown to promote the production of growth promoting phytohormones [26] such as IAA [27, 28], cytokinin [29–31] and gibberellins [32–34], inhibit the synthesis of ethylene [28, 35], and regulate endogenous ABA levels in plants [10]. Furthermore, *Serratia marcescens* strain 90–166 has been shown to protect plants against cucumber mosaic virus (CMV) in a jasmonic acid (JA)-dependent but SA/NPR1-independent manner, demonstrating the activation of a JA signaling pathway by PGPR [36, 37]. Plant hormones have also been known to act in synergistic and/or antagonistic manner to each other. For example, auxin and cytokinin act antagonistically during biotic stress [38]. Similarly, ABA-cytokinin and SA-cytokinin antagonism has been shown to modulate resistance against plant pathogens in tobacco and rice respectively [39, 40]. Thus, in addition to their plant growth promoting properties, PGPR can also provide protection against plant pathogens. Detailed reviews of the physiological impacts of PGPR and their roles in the mitigation of different abiotic and biotic stresses in plants are available in the literature [2, 4, 6–8, 22, 36].

The above information shows that PGPR are highly diverse in their plant growth promoting properties and represent a versatile target for use in improving overall crop production. For successful targeted interventions in the soil microbiome, however, it is important to consider soil microbial diversity, as there exist an intricate balance between the different types of microorganisms (and their relative populations) in the rhizosphere. Soil microbial diversity is primarily determined by the extensive communication that takes place between plant roots and associated microorganisms. This communication mostly takes place by means of the different chemical compounds secreted by plant roots and rhizospheric microorganisms, compounds that may serve as nutrients, toxins, or signaling molecules.

PGPR mainly belong to the genera *Azospirrillum*, *Azotobacter*, *Arthrobacter*, *Bacillus*, *Clostridium*, *Enterobacter*, *Pseudomonas*, and *Serratias* [41] and among these, the species of *Bacillus* and *Pseudomonas* have perhaps been the most extensively studied. *Bacillus aryabhattai* (strain B8W22) was initially, isolated from cryotubes used to collect air samples from the Earth’s stratosphere at an altitude between 27 and 41 km [42], resulting in suggestions of a cosmic origin of this bacterium. Subsequently, the bacterium was isolated from rhizosphere soil in many parts of the world such as South Korea [43], India [44], and Tibet [45]. The plant growth promoting activity of *B*. *aryabhattai* was first reported by Lee et al., who showed in 2012 that *B*. *aryabhattai* promotes the growth of *Xanthium italicum* [43]. More recently, it has been shown that zinc solubilizing strains of *B*. *aryabhattai* improved the growth of soybean and wheat plants by increasing the mobilization and bio-fortification of zinc [46]. *B*. *aryabhattai* strains have also been found useful for several other purposes, such as the biosynthesis of thermostable alkaline phosphatase [47] and the anti-leukemic tumor-inhibiting l-asparaginase enzyme [48, 49], as well as pesticide degradation [44].

Except for the above two reports, however, no information is available on the growth promoting effects of *B*. *aryabhattai* and/or its involvement in improving plant tolerance to biotic and abiotic stresses. In this study, we evaluated the plant growth promoting properties of *B*. *aryabhattai* strain SRB02 in soybean and rice plants.

## Materials and methods

### Isolation and identification of *B*. *aryabhattai*

Bacteria were isolated from the rhizosphere soil (soil attached to the roots of the plants) of a soybean field in the Chungcheong buk-do region of South Korea after prior permission from the owner of the field. For this purpose, 1 g of soil was suspended in 9 ml sterile 0.85% NaCl. The soil suspension was serially diluted up to 10^−6^, and then 100 μl of the suspension was spread on tryptic soy agar (TSA) medium (MB Cell, Los Angeles, CA, USA). Multiple single colonies were selected and sub-cultured in 5 ml tryptic soy broth (TSB) medium (MB Cell) for 24 h at 30°C. The identity of the bacterial isolates was confirmed through 16S rRNA sequencing (Solgent, Daejeon, Korea). The phylogenetic position of the bacterial isolates was determined by blasting the 16S rRNA sequence at NCBI (http://blast.ncbi.nlm.nih.gov/Blast.cgi).

### Growth of bacteria

For all the experiments, bacteria were grown either on LB-agar or LB-broth (Applichem, Darmstadt, Germany) and incubated at 30°C for 24 h. *Bacillus thuringiensis* strain ATCC 10792 (hereafter referred to as AY1) was used as a control bacterium of the same genus in subsequent experiments.

### Soybean growth conditions

Seeds of the soybean cultivar Daewon were germinated on horticultural soil (fungus-free biosoil, Dongbu Farm, Hannong, South Korea) in ¾ filled plastic pots kept in a growth chamber at 28°C /25°C (day/night), 65% relative humidity and at light intensity of 1000 μmol/m/s under long-day conditions (16 h light and 8 h dark).

### Heat stress treatment and bacterial inoculation

Soybean plants in vegetative stage 3 [(V3) the stage at which the plants are unifoliate and the first three trifoliate leaves are fully developed (https://extension.entm.purdue.edu/fieldcropsipm/soybean-stages.php) were used for the heat stress experiment at 38°C/30°C (day and night) as suggested by Li et al. [50] and others [51, 52]. For this purpose, soybean plants were grown in pots (10 cm deep and 10 cm wide at the top). Prior to the heat treatment, the soil surface was drenched with 10 ml of LB-grown bacterial culture (1 × 10^8^ cfu/ml), or with sterilized LB-broth, or with double distilled water for three days. Following a three-day acclimation period, plants were kept at 38°C /30°C (day/night) for heat stress treatment. Control plants were grown at the optimum temperature conditions mentioned above. Plants were regularly observed and data were recorded. Leaf samples were collected after 0 h, 12 h, and 48 h of heat treatment.

### Plant growth parameters

Data were recorded on different growth parameters such as shoot length (SL), root length (RL), fresh weight (FW), and dry weight (DW) after 10 days of bacterial inoculation. All data were recorded on 10 plants per replicate and the experiment was repeated 3 times. Data were statistically evaluated with Duncan’s multiple range test at a 95% level of confidence using SAS 9.1 (www.sas.com).

### Scanning electron microscopy

Scanning electron microscopy was performed to confirm bacterial colonization of the plant roots, and also to check stomatal movement in the leaves after treatment with SRB02 for analysis of heat stress-related phenotypes. For this purpose, inoculated soybean plants were up-rooted carefully, and the roots were gently washed in sterile distilled water and then freeze dried. Approximately 5 mm^2^ pieces from leaves and roots of inoculated and control plants were sputter-coated with gold using ion sputtering device (JFC-110E, EC&G, USA) and visualized with an FE-SEM scanning electron microscope (S-4300, Hitachi, Tokyo, Japan).

### Quantitative measurement of hormones from bacteria

#### Abscisic Acid (ABA) measurement

Bacterial ABA measurement was performed as described by Cohen et al. [53]. For this purpose, 20 ml of extraction solution consisting of 99% EtOAc (ethyl acetate), 1% glacial acetic acid, and 20 ng of the internal standard [(±)-3,5,5,7,7,7-d6]-ABA, was added to 10 ml of bacterial culture media. The aqueous phase was collected and extracts were evaporated. Dried residues were re-suspended in phosphate buffer (pH 8.0), passed to polyvinylpolypyrrolidone (PVPP) columns with pH adjusted to 3.5 using 6N HCl and again then partitioned three times with EtOAc. Extracts were then combined and evaporated. Dried residues were re-suspended in dichloromethane (CH_2_Cl_2_) and passed through a silica cartridge (Waters, Milford, MA, USA) pre-washed with 10 ml of diethyl ether:methanol (3:2 v/v). The extracts were dried again and methylated by adding diazomethane for ABA analysis in a GC/MS-SIM [Gas Chromatography/Mass Spectrometry (GC/MS)–Selected Ion Monitoring (SIM)] (6890N network GC system and 5973 network mass selective detector; Agilent, Palo Alto, CA, USA) and quantified using Lab-Base software (Thermo Quest, Manchester, UK).

#### Indole Acetic Acid (IAA) measurement

Preliminary tests were performed for the presence of IAA in bacterial cultures using Salkowski reagent [54–56]. Bacterial IAA measurements were then performed following the method of Park et al. [57]. Briefly, 10ml bacterial cultures were grown at 30°C for 2 days. Uniform concentrations of bacterial culture were obtained by measuring the optical density of bacterial cultures at 600 nm and centrifuged at 15,000 rpm for 2 min. The supernatants were filtered through a 0.45 μm cellulose acetate filter and the pH was adjusted to 2.8 with HCl before adding 40 μl of [D5]-IAA as an internal standard. Extracts were then partitioned with 4 ml ethyl acetate. The aqueous phases were combined together and evaporated under a vacuum at 45°C. Dried residues were re-suspended using 60% methanol and the pH was adjusted to 8 ± 0.3 using 2N NH_4_OH, and the suspension was passed through a reverse-phase C18 column. The methanolic fractions were prepared by dissolving the residue in 1 ml of methanol and adding 1.5 ml ethereal diazomethane. The methylated samples were re-dissolved in ethyl acetate before the IAA analysis using GC—MS with SIM (6890 N network GC system, and 5973 network mass selective detector; Agilent, Palo Alto, CA, USA).

#### Gibberellic Acid (GA) measurement

The quantification of GA in the bacterial cultures was carried out according the protocol described by Lee et al. [58]. Bacterial culture filtrates supplemented with (^2^H_2_) GA standards were processed for detection, identification and quantification of GAs using gas chromatography and mass spectroscopy.

### Quantitative measurement of plant endogenous ABA, JA, SA, GA, IAA and cytokinin contents

Freeze-dried plant samples finely ground with a mortar and pestle were used for all the endogenous plant hormone analyses. ABA measurement was performed following the method of Qi et al. [59]. Briefly, 0.1 g plant samples were dissolved in 20 ng of the internal standard [(±)-3,5,5,7,7,7-d6]-ABA, including isopropanol:acetic acid (95:5) extraction solution, and evaporated. They were dissolved again into 5 ml of 1N NaOH and washed three times with 3 ml of dichloromethane to remove chlorophyll. The aqueous phase was collected, pH was adjusted to 3.5 with 6 N HCl, and the samples were partitioned three times in 10 ml ethyl acetate (EtOAc) and evaporated. Residues were dissolved in phosphate buffer (pH 8.0) and run through a polyvinyl polypyrrolidone (PVPP) column. After passing through the column, the pH of the extracts was adjusted to 3.5 with 6N HCl, and the samples were again partitioned three times with EtOAc. The extracts were evaporated and methylated with diazomethane and then GC/MS-SIM analysis was conducted (6890 N network GC system, and the 5973 network mass-selective detector; Agilent, Palo Alto, CA, USA). For quantification, Lab-Base software was used (ThermoQuest, Manchester, UK).

JA concentrations were measured following the method of Creelman and Mullet [60]. Briefly, 0.1 g plant powder was suspended in an extraction solution of acetone:50 mM citric acid (70:30, v/v) and 20 ng of (9,10-2H2)-9,10-dihydro-JA was added as an internal standard. The suspension was evaporated at room temperature overnight and re-suspended in 0.1 M potassium phosphate buffer, and the pH was adjusted to 2.5. Chlorophyll was removed from the samples using diethyl amino ethyl cellulose (DEAE-cellulose) and then partitioned using chloroform. Samples were then filtered through Na_2_SO_4_ and evaporated. Extracts were re-dissolved in 5 ml of diethyl ether three times then purified by passing them through a solid phase extraction cartridge (500 mg of sorbent, amino propyl). The cartridges were washed with 5 ml of chloromethane and 2-propanol solution (2:1, v/v) three times. JA was then eluted three times with 2 ml of diethyl ether and acetic acid solution (98:2, v/v) before methylation. The extracts were then analyzed by GC-MS as described above.

Salicylic acid (SA) in SRB02-treated soybean plants was extracted as described by Seskar et al. [61]. High performance liquid chromatography (HPLC) was performed using a C18 reverse phase column (HP Hypersil ODS, particle size 5 mm, pore size 120 Å; Waters, Milford, MA, USA) and a Shimadzu RF-10AZL (Shimadzu, Kyoto, Japan) equipped with a fluorescence detector with excitation and emission set at 305 nm and 365 nm, respectively. Flow rate was maintained at 1.0 ml/min. For plant endogenous GA measurement, samples were lyophilized and ground into fine powder using a mortar and pestle. Afterwards, samples were processed as described above for bacterial GA measurements.

Plant IAA and cytokinin (Trans-Zeatin) measurements were performed as described by [62] and [63] with minor modifications using Liquid Chromatography Electrospray-Ionization Quadrupole Time-of-flight (LC-ESI-QTOF) Tandem Mass Spectrometry. Briefly, 200 mg of freeze dried leaf samples from treated and un-treated control plants were finely ground and 2 ml of extraction buffer [2-propanol: H_2_O: concentrated HCl (2:1:0.002, vol/vol/vol)] was added and incubated at 4°C on a shaker for 30 min. Then 2 ml of dichloromethane was added to each sample and incubated at 4°C on a shaker for 30 min before centrifugation at 13,000g for 5 min at 4°C. The lower phase was then transferred to another tube and concentrated using nitrogen evaporation and re-dissolved in 0.1 ml methanol. Finally 10 μl of the resulting solution was analyzed in a XEVO G2-S QTOF Spectrometer (Waters, USA) connected with a UPLC system (Waters, USA) using Acquity UPLC BEH C18 1.7 μM (2.1 x 100 mm) column. All the experiments were repeated at least three times.

Quantitative measurements for all plant and bacterial hormones were performed three times. Data were subjected for analysis of variance (ANOVA) using SigmaPlot^®^ (10.0). Mean values were compared using Duncan’s Multiple Range Test (DMRT) at 95% level of significance.

### Stress media tests

*B*. *aryabhattai* SRB02 were tested for tolerance to multiple oxidative and nitrosative stresses. For this purpose, SRB02 were grown on oxidative and nitrosative stress media. Oxidative stress was given by supplementing LB agar media with 0.75 μM, 1 μM, 1.5 μM and 2 μM methyl viologen (MV) and 50 μM, 100 μM, 200 μM and 500 μM hydrogen peroxide (H_2_O_2_), while nitrosative stress was given by supplementing LB agar media with 4 mM, 4.5 mM, 5 mM and 6 mM S-nitrosoglutathione (GSNO) and 3.5 mM, 4 mM, and 5 mM S-nitrocysteine (CysNO). Pilot experiments were first conducted to adjust the above optimum concentrations. *B*. *thuringiensis* strain AY1 was used as a control.

### SOD-like activity test

The results of the stress media showed that SRB02 tolerated high oxidative stress given by both MV (donor of the superoxide O˙ˉ_2_ radical) and H_2_O_2_. This prompted us to investigate the superoxide dismutase (SOD) and catalase activities of these bacteria. An SOD activity test was performed following established procedures [64, 65] with minor modifications. For this purpose, 3 ml of 50 mM Tris-cacodylic acid buffer (TCB, pH 8.2) was added to 2 ml LB bacterial cultures (O.D 600nm = 0.2) and centrifuged at 8000 rpm for 5 min. Next, 950 μl of the supernatant was mixed with 50 μl of 10 mM pyrogallol (A). Bacterial supernatant with TCB (B), TCB with pyrogallol (C), and sterilized LB broth with pyrogallol, were used as comparative controls. The experiment was repeated three times and data were analyzed for significant differences through Student’s T-test at 95% level of significance using Microsoft Excel.

Pyrogallol (1,2,3-benzenetriol) is known for its high rate of autoxidation, especially at higher pH values, and it has been used for separation of oxygen from other gases. SOD enzyme rapidly dismutases univalent reduced oxygen, also called the superoxide anion radical (O˙ˉ_2_). SOD reduces the autoxidation of pyrogallol by about 99% in the presence of the Oˉ_2_˙ anion radical [66]. Hence, the extent of SOD activity can be estimated from the ability of the SOD enzyme to inhibit the autoxidation of pyrogallol which is measured in terms of changes in the absorbance (A) at a wavelength of 420 nm overtime at 25°C. We measured this change in absorbance after 2 min, 5 min, and 10 min, and we calculated SOD-like activity by the following formula [65].

$$SOD\;activity\;\left(\%\right)=1-\left(\frac{A-B}{C}\right)\times 100$$

### Catalase activity test

Catalase activity was tested using an Amplex^®^ Red Catalase Assay Kit (Molecular Probes, Thermo Fisher, Waltham, MA, USA) according to the manufacturer’s instructions. The kit provides an ultrasensitive but, simple method for measuring catalase activity based on a competition assay between catalase enzyme and the Amplex Red reagent. Catalase first reacts with H_2_O_2_ producing H_2_O and O_2_. The Amplex Red then reacts with any remaining H_2_O_2_ in the presence of horseradish peroxidase (HRP), producing a fluorescent oxidation product, resorufin. Increased production of catalase enzyme(s) overtime reduces the resorufin signal. Overnight bacterial cultures (O.D 600 = 0.2) were centrifuged and the pellets were lysed in 0.1 M Tris buffer (pH 7.4).

The experiment was performed in a 96-well plate by adding 25 μl each of SRB02 and AY1 cell lysates, catalase standard curve samples, and 40 μM H_2_O_2_. Following the decomposition of hydrogen peroxide by catalase, 50 μM Amplex Red reagent and 0.2 U/ml horseradish peroxidase (HRP) were added. Fluorescence was measured using a Multiskan^™^ GO microplate spectrophotometer (Thermo Fisher, Waltham, MA, USA) at 590 nm. The experiment was repeated three times and data were analyzed for significant differences through Student’s T-test at 95% level of significance using Microsoft Excel.

### Gene expression analysis

Total RNA was extracted using TRIzol reagent (Invitrogen, Carlsbad, CA, USA) and subsequent ethanol precipitation before cDNA synthesis. One microgram of RNA was used to synthesize cDNA using a DiaStarTM RT Kit (Solgent, Daejeon, South Korea). Gene expression was analyzed by two-step quantitative real time PCR using a QuantiSpeed SYBR Green Kit (PhileKorea, Seoul, South Korea) in an Eco^™^ real-time PCR machine (Illumina, San Diego, CA, USA) for 40 cycles. Each reaction contained 10 μl master mix, 1 μl each of 10 pmol/μl primers (S1 Table), and 1 μl of cDNA, with final volume adjusted to 20 μl using nuclease free water. The PCR reaction was performed with an initial denaturation step at 95°C for 2 min and subsequent denaturation steps at 95°C for 10 s followed by annealing and extension at 60°C for 30 s for 40 cycles.

## Results

### *B aryabhattai* SRB02 as a plant growth promoting bacterium

As described above, we isolated bacteria from the rhizosphere soil of a soybean field in the Chungcheong buk-do region of South Korea. After obtaining pure cultures, the target bacterium was identified through 16S rRNA sequencing. The phylogenetic position of the bacterial isolate was determined by blasting the 16S rRNA sequence on NCBI (http://blast.ncbi.nlm.nih.gov/Blast.cgi). Phylogenetic analysis showed 100% similarity of the 16S rRNA sequence of our target bacterium to *B*. *aryabhattai* strain SRB02 (S1 Fig) first reported by Kang et al. from South Korea (NCBI accession No. KP860638.1) (https://www.ncbi.nlm.nih.gov/nucleotide/803432357?report=genbank&log$=nuclalign&blast_rank=1&RID=RW73JGVC01R).

**Fig 1:**
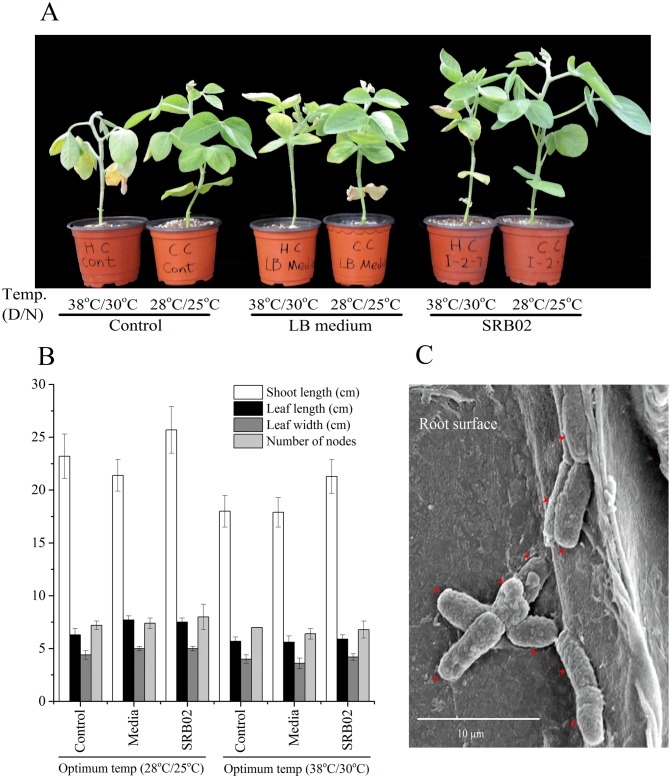
*Bacillus aryabhattai* SRB02 as a plant growth promoting rhizobacterium. Soil-grown soybean plants in vegetative stage 3 (V3) were inoculated (by soil drenching) with LB-grown bacterial culture (1 × 10^8^ cfu/ml), or with sterilized LB broth or with double distilled water for 3 days and kept at 38°C/30°C (day/night) for the heat stress treatment, following a 3-day acclimation period (A). SRB02 promoted the growth of soybean plants under all temperature regimes and significantly increased the shoot length of soybean plants as well as leaf length, leaf width and number of nodes (B) after successful colonization of roots within 2 days of inoculation (C). Each data point in panel B represents the mean of at least three replicates. Error bars represent standard deviations. Bars with different letters are significantly different from each other at P ≤ 0.05.

The overall analyses performed in this study showed that *B*. *aryabhattai* strain SRB02 (hereafter referred to as SRB02) significantly promoted the growth of soybean and rice. Scanning electron microscopy showed that SRB02 successfully colonized the roots of soil-grown soybean plants within 2 days of inoculation (Fig 1C) and resulted in significantly greater shoot length, leaf size, and node number of both soybean (Fig 1A and 1B) than those observed for control treatments, indicating that SRB02 is a plant growth promoting bacterium. In soybean plants in particular, the growth promoting effect was observed in plants grown under optimum as well as under high temperature conditions, indicating that SRB02 ameliorates heat stress in soybean.

### *B*. *aryabhattai* SRB02 regulates the production of growth hormones in plants

SRB02-mediated growth promotion of soybean and rice plants prompted us to investigate whether SRB02 can produce growth hormones and/or affect the regulation of endogenous phytohormones. For this purpose, we measured various phytohormones in bacterial cultures as well as in plants treated with SRB02.

### SRB02 produces ABA and helps plants maintain ABA levels during heat stress

**Fig 2:**
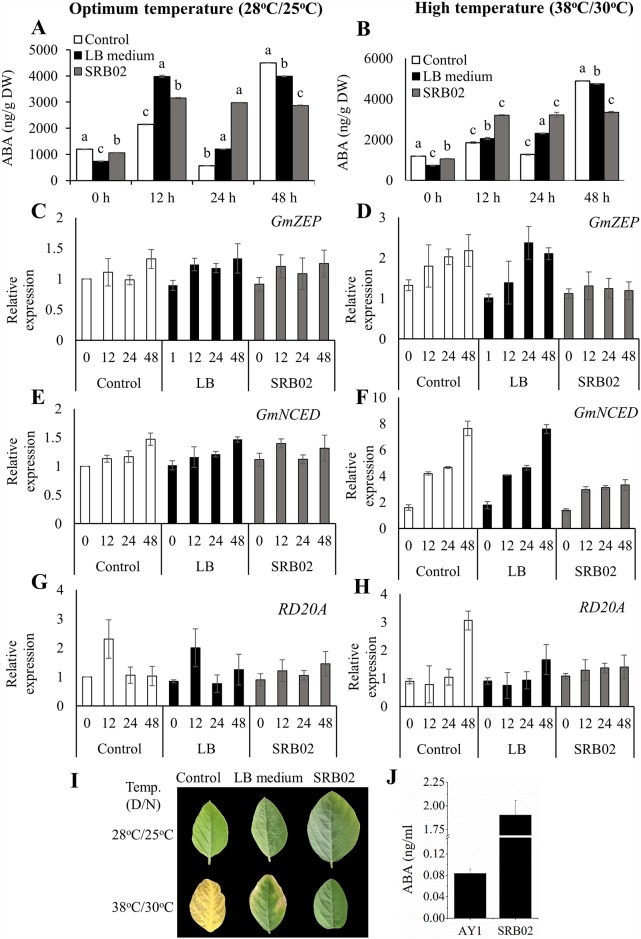
*Bacillus aryabhattai* SRB02 produces ABA and regulates its levels in plants. Plants treated with *B*. *aryabhattai* SRB02 produced constant levels of ABA under normal temperature conditions (A) as well as under heat stress (B). These results are consistent with the very similar transcript accumulation of the ABA-related genes *ZEP* (C, D), *NCED* (E, F), and *RD20A* (G, H) from 12 h to 48 h of SRB02 treatment under both temperature regimes. Plants treated with SRB02 were more tolerant of heat stress and remained green, while the leaves of control plants showed chlorotic symptoms (I). SRB02 produced a significantly higher amount of ABA than did AY1 (J). Each data point represents the mean of at least three replicates. Error bars represent standard deviations. Bars with different letters are significantly different from each other at P ≤ 0.05.

Plants treated with SRB02 showed a sustained production of ABA from 12 h to 48 h after inoculation under both temperature regimes (Fig 2A and 2B). Similar results were obtained using qRT-PCR analysis of genes involved in ABA biosynthesis: *GmZEP* (Glyma01g39310), *GmNCED* (Glyma05g27250), and ABA responsive gene *GmRD20A* (Glyma03g41030). Very similar transcript levels were recorded for these genes in soybean plants treated with SRB02 12 h to 48 h after inoculation, especially in plants grown at high temperature (Fig 2C–2H).

Interestingly, we observed medium to severe leaf chlorosis in control soybean plants following heat stress treatment (Fig 2I), and increased expression of ABA biosynthesis genes (Fig 2D, 2F and 2H). On the other hand, in SRB02-treated plants, which produced normal levels of ABA after 12 h to 24 h after inoculation, no chlorosis was observed (Fig 2I). Analysis of bacterial cultures showed that SRB02 produced up to 2 ng/ml ABA in culture (Fig 2J). ABA-mediated drought stress tolerance through stomatal closure is well known and as we expected, the stomata of plants treated with SRB02 were almost closed under high day/night temperatures of 38°C/30°C compared to those of control plants (S2 Fig). Analysis of SRB02 cultures showed at least 20-fold higher ABA accumulation than that of the control bacterium *B*. *thuringiensis* strain AY1 (Fig 2J).

### SRB02 produces IAA and modulates auxin signaling in soybean plants

**Fig 3:**
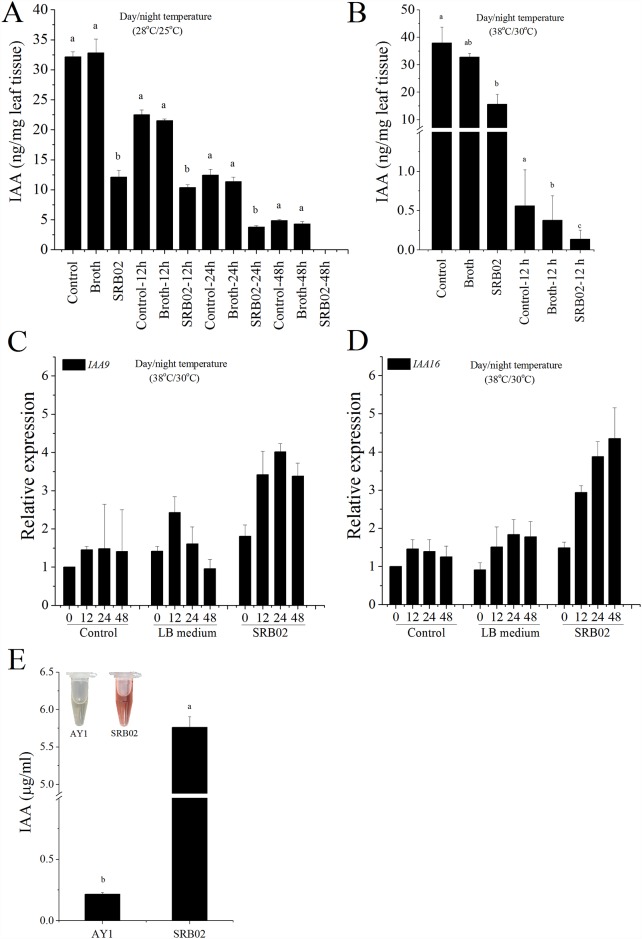
*Bacillus aryabhattai* SRB02 produces IAA and transcriptionally regulates its production in plants. Soybean plants treated with SRB02 accumulated lower quantity of IAA under optimum (A) and heat stress conditions (B). SRB02-treated plants showed significantly higher expression of the Aux/IAA transcriptional repressor genes *IAA9* (C) and *IAA16* (D) genes. Preliminary tests with Salkowski reagent showed the production of IAA in SRB02 culture (Inset E). GC-MS analysis showed that SRB02 produced significantly higher amounts of IAA than did AY1 (E). Error bars represent standard deviations. Each data point represents the mean of at least three replications. Bars with different letters are significantly different from each other at P ≤ 0.05.

SRB02-treated soybean plants showed low IAA levels compared to untreated control plants at all the time points (Fig 3A). SRB02-mediated low IAA levels were observed in plants grown at both normal and high temperature conditions. Furthermore, in plants grown under heat stress conditions significant quantity of IAA could only be detected up to 12 h after SRB02 treatment (Fig 3B). Quantitative real time PCR analysis of plants grown under heat stress showed significantly higher accumulation of two genes involved in auxin signaling: *GmIAA16* (Glyma02g16071) and *GmIAA9* (Glyma01g02350) (Fig 3C & 3D) which both encode the Aux/IAA transcriptional repressor proteins [67, 68] which explains low accumulation of IAA in SRB02-treated plants. Analysis of SRB02 broth cultures with van Urk—Salkowski reagent indicated high concentrations of auxin derivatives (Inset, Fig 3E). Salkowski reagent is a chromogenic reagent having high sensitivity and specificity to approximately 80 different indoles, and it gives a pinkish to orange color upon reaction. Further chromatographic analyses of SRB02 and AY1 cultures using GC-MS showed a more than 20-fold increase in IAA in SRB cultures over that in AY1 cultures (Fig 3E).

### SRB02 modulates JA biosynthesis and cross-talk with SA in soybean

**Fig 4:**
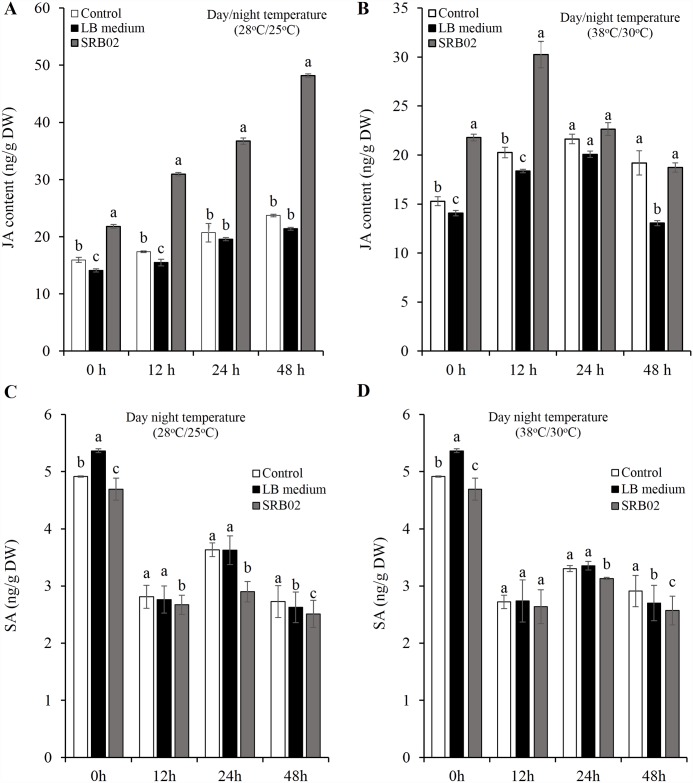
*Bacillus aryabhattai* SRB02 promotes the production of jasmonic acid in plants. SRB02-treated soybean plants produced significantly higher quantities of jasmonic acid under optimum temperature conditions at all the time points studied (A). Though JA accumulation gradually declined from 12 h to 48 h after inoculation with SRB02 under heat stress, JA content was significantly higher in SRB02-treated plants than in un-treated control plants for up to 12 h after inoculation (B). SRB02-treated soybean plants showed significantly less SA accumulation after 12, 24 and 48 h of inoculation both at optimal temperature (C) and high temperature conditions (D). Error bars represent standard deviations. Each data point represents the mean of at least three replications. Bars with different letters are significantly different from each other at P ≤ 0.05.

Analysis of soybean plants revealed a rapid increase in the biosynthesis of JA within 12 h of inoculation and continuing up to 48 h (Fig 4A). However, under heat stress conditions, JA levels decreased drastically after 12 h of SRB02 treatment (Fig 4B), though the concentration of JA in SRB02-treated plants was still higher than in the control plants. Endogenous JA levels in plants decrease significantly within the first 12 h of heat stress treatment accompanied by the suppression of genes involved in JA biosynthesis [69]. As a result of the marked increase in JA levels caused by SRB02 in soybean plants grown under optimum temperature conditions, we expected JA levels to at least remain constant after heat treatment in SRB02-treated plants. However, to our surprise, JA levels declined significantly after 12 h of inoculation with SRB02 under heat stress indicating that SRB02-mediated activation of the JA pathway is dependent upon growth conditions. Several studies have reported PGPR-mediated protection of plants through the activation of ISR, a defense pathway regulated by JA. Our results indicated that following SRB02-mediated increase in JA content, the SA levels remained almost constant at all the time points under study (Fig 4C and 4D), indicating that root colonization by SRB02 does not activate an SA dependent defense response.

### SRB02 produces GAs and regulates GA biosynthesis in soybean

**Fig 5:**
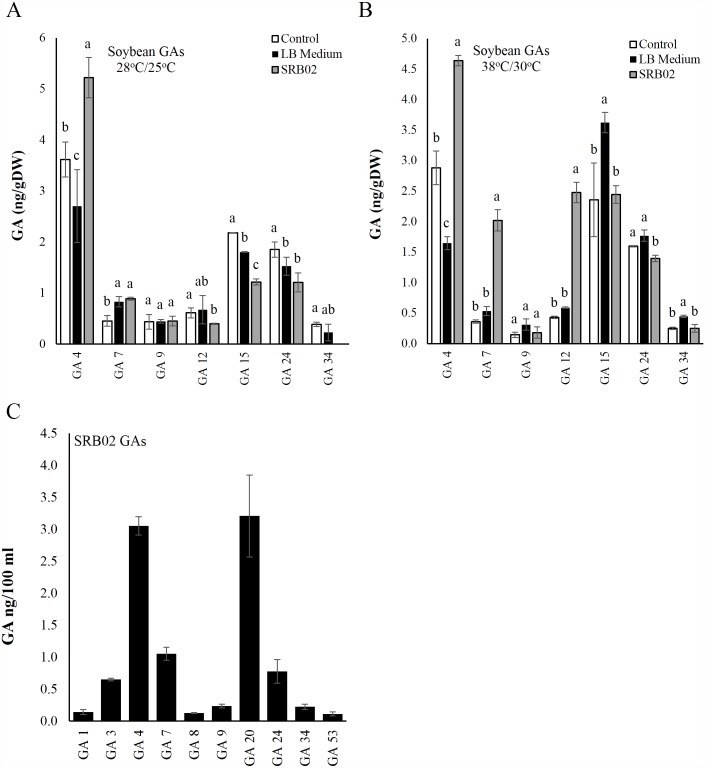
*Bacillus aryabhattai* SRB02 produces gibberellins and regulates their production in soybean. GC-MS analysis of plants SRB02-treated plants grown under normal temperature conditions did not show significant variation in the accumulation of different GA derivatives except for GA4 and GA7 (A). Under high temperature conditions, significantly higher quantities of GA4, GA7 and GA12 were recorded (B). Analysis of bacterial cultures revealed significant quantities of several GA derivatives (C). Error bars represent standard deviations. Each data point represents the mean of at least three replications. Bars with different letters are significantly different from each other at P ≤ 0.05.

Analysis of SRB02-treated soybean plants grown under normal temperature conditions did not show a significant increase in the concentration of most GAs except for GA4 and GA7 (Fig 5A). However, SRB02-treated plants grown under heat stress showed markedly greater production of several GA derivatives than that in control plants (Fig 5B). SRB02 produced the GA derivatives GA1, GA3, GA4, GA7, GA8, GA9, GA20, GA24, GA34, and GA53, with the highest quantities being for GA4 and GA20, followed by GA7, GA24, and GA3 (Fig 5C).

### SRB02 produces cytokinin and regulates cytokinin homeostasis in soybean

**Fig 6:**
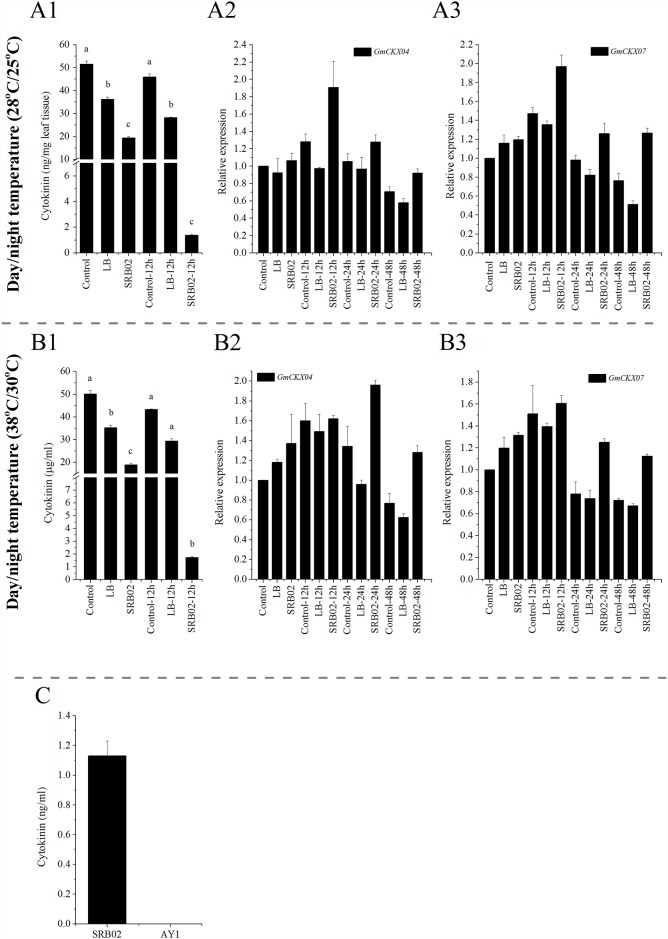
*Bacillus aryabhattai* SRB02 produces cytokinin and regulates cytokinin homeostasis in soybean. Results showed a significant and rapid reduction in cytokinin levels of SRB02-treated soybean plants grown under normal temperature conditions after 12 h of inoculation (A1) potentiated by significantly higher transcript accumulation of the cytokinin-dehydrogenase genes *GmCKX04* (A2) and *GmCKX07* (A3). Cytokinin levels also decreased after treatment of soybean plants with SRB02 under heat stress conditions (B1) with a concomitant increase in the expression of *GmCKX04* (B2) and *GmCKX07* (B3) genes. LC-ESI-QTOF analysis showed that SRB02 produced about 1.5 ng/ml cytokinin in liquid culture (C) compared to AY1 which did not produce any cytokinin. Each data point represents the mean of at least three replicates. Error bars indicate standard deviation. Bars with different letters are significantly different from each other at P ≤ 0.05.

As described earlier, the antagonistic behavior of auxin and cytokinin is well known. The SRB02 mediated perturbation of auxin levels as well as of the auxin related transcriptional machinery in soybean plants under normal and heat stress conditions tempted us to investigate cytokinin levels in plants following SRB02 application. Results showed a significant and rapid reduction in cytokinin levels following SRB02 treatment under both temperature regimes. The least quantity of cytokinin was recorded after 12 h of SRB02 treatment whereas no cytokinin could be detected later in the plants treated with SRB02 whether grown under optimum temperature conditions or under heat stress (Fig 6A1 & 6B1, respectively). Quantitative real time PCR analysis showed a marked increase in the transcript accumulation of two genes *GMCKX04* and *GmCKX07* in SRB02-treated plants grown under normal temperature (Fig 6A2 & 6A3) and high temperature conditions (Fig 6B2 & 6B3). The *CKX* is a multi-gene family present throughout the plant kingdom encoding cytokinin dehydrogenase proteins that irreversibly degrade the phytohormone cytokinin in plants [70, 71]. LC-ESI-Q-TOF analysis showed that SRB02 produced about 1.5 ng/ml cytokinin in liquid culture (Fig 6C).

### *B*. *aryabhattai* tolerates high oxidative and nitrosative stress

**Fig 7:**
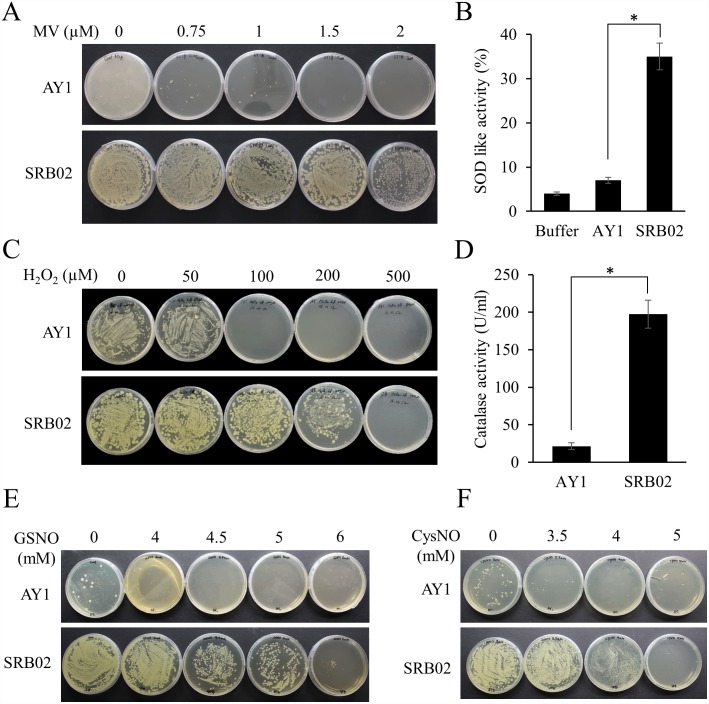
*Bacillus aryabhattai* SRB02 tolerates high oxidative stress and nitrosative stress. SRB02 was found to be significantly more tolerant of oxidative stress provoked by up to 2 μM MV than was AY1 (A), potentiated by significantly higher SOD activity (B). SRB02 was also significantly more resistant to H_2_O_2_ than was AY1 (C) due to significantly high catalase activity (D). SRB02 was significantly more tolerant of nitrosative stress induced by various concentrations of the NO donor GSNO (E) and CysNO (F) than AY1. Error bars represent standard deviations. Each data point represents the mean of at least three replications. Bars with asterisk are significantly different (Student’s t-test) from each other at P ≤ 0.05.

Attachment, entry, or colonization of plant roots or any of the above-ground parts of a plant by a pathogen usually triggers a defense response. Whether the defense response is successful or not is the outcome of highly complex plant-pathogen interactions. Plant defense responses are most often characterized by a change in the cellular redox state with an increased accumulation of reactive oxygen species (ROS) and/or reactive nitrogen species (RNS). Successful SRB02 root colonization and growth promotion of soybean by regulation of phytohormone homeostasis prompted us to investigate the response of these bacteria to oxidative and nitrosative stresses. For this purpose, we grew *B*. *aryabhattai* SRB02 cultures on LB medium containing various concentrations of MV and H_2_O_2_ (for oxidative stress) and the nitric oxide donors GSNO and CysNO (for nitrosative stress). Interestingly, SRB02 was able to tolerate the high oxidative and nitrosative stresses induced by all the treatments better than AY1. SRB02 grew efficiently on LB-agar plates containing up to 2 μM MV and 200 μM H_2_O_2_. Analysis of SRB02 cultures indicated significantly higher SOD (Fig 7A and 7B) and CAT activities than observed for AY1 (Fig 7C and 7D), indicating the presence of highly active and efficient antioxidant machinery in SRB02. Similarly, SRB02 also grew much better on GSNO and CysNO plates (Fig 7E and 7F).

## Discussion

The use of PGPR as microbial inoculants is becoming a widely accepted practice in intensive agriculture, especially in the developed countries [72]. For this purpose, aggressive colonization of plant roots is one of the key criteria used for selection of efficient PGPR. Root colonization by various PGPR has been reported to occur in one to a few weeks, with bacterial populations reaching a maximum point within 60 days [72]. The successful soybean root colonization by SRB02 within 2 days of inoculation was surprisingly fast. This rapid root colonization may well be aided by the response of SRB02 to exudates from soybean roots. Another plant growth promoting bacterium from the genus *Bacillus* that responds to root exudates is *B*. *amyloliquefaciens*. Strain NJN-6 of this bacterium has been shown to respond to organic acids from root exudates of banana [73]. PGPR-mediated growth promotion is typically associated with enhanced production of growth-related phytohormones such as auxin, ABA, cytokinin, GA, and others though, it is now generally agreed that the phytohormones produced by PGPR may not be of any physiological importance to these bacteria *per se* but are instead produced by them as secondary metabolites in order to establish a better association with their host plants.

Since ABA and GA biosynthesis follow the same pathway in plastids through the non-mevlonate or methylerythritol phosphate (MEP) biosynthetic route of terpenes [74], one feasible hypothesis is that PGPR can produce both ABA and GA simultaneously and possibly enhance the ABA and GA levels of plant hosts. Adaptive responses of plants to heat stress usually include an increase in endogenous ABA levels [75]. The effect of different concentrations of ABA on heat stress tolerance of different plants has been extensively studied [76, 77]. However, continuous production of high ABA levels is toxic to plants and leads to leaf chlorosis/necrosis [78–80]. Interestingly, we observed medium to severe leaf chlorosis in control soybean plants following heat stress treatment (Fig 2I), However, this may also be the effect of ABA accumulation or ABA induced ethylene production following heat stress treatment [81, 82]. The *B*. *aryabhattai* strain SRB02 isolated in this study not only produced ABA and GA but also significantly enhanced the levels of these phytohormones in soybean plants.

Abscisic acid positively regulates drought stress tolerance in plants [83]. ABA-mediated drought stress tolerance through stomatal closure is well known [84]. Since different types of stresses induce the production of ABA in plants, it is now widely regarded as a stress hormone and its physiological impact and its production under different basal and induced conditions has been described [84–86]. SRB02-mediated heat stress tolerance of soybean plants potentiated through sustained production of ABA and expression of related genes compelled us to investigate stomatal movement in soybean plants under heat stress. As expected, the stomata of plants treated with SRB02 were almost closed under high day/night temperatures of 38°C/30°C compared to those of control plants (S2 Fig). These results are similar to the findings of other studies that reported the production of ABA by different PGPR, as well as enhancement of the levels of ABA and other phytohormones in host plants. In 2001 and 2007, Cohen et al. demonstrated that *Azospirillum lipoferum* and *A*. *brasilense* Sp. 245 not only produce ABA but they also enhance ABA levels in maize and *Arabidopsis* plants 2-fold [53, 87, 88]. By comparison, the *B*. *aryabhattai* strain SRB02 identified in this study produced up to 2 ng/ml ABA and enhanced ABA levels of soybean plants more than 4-fold especially at the earlier time points of 12 h post inoculation.

It appears that plants synthesize ABA through cleavage of carotenes such as neoxanthin [89], while lower organisms such as fungi produce it directly from farnesyl pyrophosphate [90]. The ABA biosynthetic pathway in bacteria is unknown, however. PGPR-mediated growth regulation is partly governed by the regulation of auxin biosynthesis. Although a very large proportion (almost 80%) of bacteria colonizing the rhizosphere have been identified as positive for IAA production, an equally large percentage of these are gram-negative, a common characteristic of phyto-pathogenic bacteria. A limited number of gram-positive soil-living bacteria have been reported to produce IAA, e.g., *B*. *subtilis* and *B*. *amyloliquefaciens*, and the first of these is also a well-established bio-control agent [91]. *B*. *aryabhattai* strain SRB02 isolated in this study produced up to 5.8 μg/ml IAA in culture. However, to our surprise, SRB02 inoculation resulted in a significant decrease in the IAA content of soybean plants grown under normal as well as heat stress conditions. Furthermore, after 48 h of SRB02 inoculation, we could not detected any IAA in soybean plants grown under optimum temperature conditions.

Modulation in phytohormone production in response to different biotic and abiotic stresses is well known. Key phytohormones such as auxin and cytokinin have been shown to decrease under heat stress [92–94]. Several plant growth promoting rhizobacterial species not only produce auxins *per se* but also regulate IAA biosynthesis in their respective host plants. At least 20 different strains of *A*. *brasilense* and *A*. *lipoferum* have been shown to produce indole-3-acetic acid (IAA), indole-3-ethanol, indole-3-methanol, and indole-3-lactic acid, and several of these strains also promoted IAA biosynthesis in maize seedlings [95]. Un-like ABA, the biosynthesis of IAA in bacteria is relatively well known, with tryptophan being the major precursor for IAA production, and with tryptophan-deficient mutants that lack *trp* gene being unable to produce IAA [28]. As such, multiple pathways have been suggested for the synthesis of IAA in bacteria, involving indole-3-pyruvic acid (IPA), indole-3-acetamide (IAM), and indole-3-acetonitrile (IAN) as important intermediates, although all use tryptophan as a precursor [96, 97]. Several PGPR have been shown to produce auxin in culture and interfere with auxin biosynthesis in plants. PGPR produce IAA from tryptophan which is present in plant roots in varying concentrations depending upon plant genotype [98]. It is also important to mention that the function of auxin in plants is concentration dependent i.e. low concentrations of IAA simulates elongation of the primary root, whereas high levels of IAA stimulate the formation of lateral roots and root hairs [99, 100]. SRB02-treated soybean plants produced more secondary roots under both temperature regimes compared to un-treated control plants (S3 Fig). This indicates that the significantly higher quantity of IAA produced by SRB02 once transported into the plant system, exerted an inhibitory effect on auxin biosynthesis and impede primary root formation. Furthermore, this higher quantity of IAA cued a counter-response measure in plant cells switching off the endogenous auxin-production machinery at transcriptional level as shown by a significant increase in the expression of transcriptional repressor genes *IAA9* and *IAA16*. During root colonization, such PGPR also produce secondary metabolites and signaling molecules such as 2,4-diacetylphloroglucinol (DAPG) and nitric oxide (NO) [101, 102]. NO has been shown to regulate auxin biosynthesis in plants [103] as well as PGPR-mediated auxin production in plants [102]. The extremely resistant nature of SRB02 to nitrosative and oxidative stress may correlate with SRB02-mediated homeostasis of IAA in soybean plants. IAA and ABA have been shown to act antagonistically *in planta* [104–106]. SRB02-mediated reduction of IAA in soybean plants may just be the antagonistic outcome of the ABA-stabilizing effect of SRB02 in these plants. Astonishingly, the production of IAA by PGPR may not always prove beneficial for plants. For example, IAA production by *Pseudomonas putida* favors root colonization of *Azospirillum brasilense*, ultimately promoting plant growth [107], but IAA production has also been found necessary for pathogenesis [108, 109]. This suggests that IAA and other plant hormones not only control plant development but can also act as signaling molecules. Furthermore, as a result of the constant interaction between plants and pathogens during the course of evolution, phyto-pathogens have learned to produce and use these signaling molecules to their advantage while communicating with other microorganisms and/or with host plants.

PGPR have been shown to protect plants against plant pathogens through a pathway that is SA and NPR1-independent but JA dependent [37]. The PGPR *S*. *marcescens* strain 90–166 induced the expression of *PDF1*.*2*::*GUS* in *Arabidopsis* Col-0 and *npr1-1* mutant backgrounds after 3 days of inoculation but did not induce *PR1* expression. It also protected SA-deficient *nahG* plants against infection by cucumber mosaic virus (CMV) [37], indicating that PGPR play an important role in induced systemic resistance (ISR). Even though strain 90–166 has been shown to produce SA it was not the primary determinant responsible for activating ISR in cucumber and tobacco against bacterial and fungal pathogens [110, 111], since the SA-negative *S*. *marcescens* strain 90-166-1441 also induced resistance against bacterial pathogens in tobacco wild type and *nahG* mutant lines [110] and in *Arabidopsis nahG* plants [37], indicating that activation of plant defense responses by some PGPR can occur only through the activation of the JA pathway but not the SA pathway. On the other hand, some PGPR may also induce systemic acquired resistance (SAR) which requires the activation of the SA pathway [112]. The *B*. *aryabhattai* SRB02 strain isolated in this study significantly enhanced JA production in soybean plants. Endogenous JA levels in plants decrease significantly within the first 12 h of heat stress treatment accompanied by the suppression of genes involved in JA biosynthesis [69]. As a result of the marked increase in JA levels caused by SRB02 in soybean plants grown under optimum temperature conditions, we expected JA levels to at least remain constant after heat treatment in SRB02-treated plants. However, to our surprise, JA levels declined significantly after 12 h of inoculation with SRB02 under heat stress indicating that SRB02-mediated activation of the JA pathway is dependent upon growth conditions.

Several PGPR-plant interactions have been shown to involve cytokinin production [29, 30, 113, 114]. It appears that PGPR-produced cytokinin mediated growth promotion in plants requires the function of cytokinin receptors *CRE1*, *AHK2* and *AHK3*, and *RPN12* a gene involved in cytokinin signaling as PGPR-mediated growth promotion was reduced in *AHK2-2* single and double mutant combinations and in *RPN12*, whereas the triple mutant *CRE1-12/AHK2-2/AHK3-3* was insensitive to PGPR inoculation [30]. In a recently published study, Grosskinsky et al. demonstrated the role of microbial cytokinin production in plant defense against the hemi-biotrophic bacterium *Pseudomonas syringae* [115]. They showed that the resistance of Arabidopsis plants to bacterial infection was potentiated by the cytokinin produced by *Pseudomonas fluorescens* strain G20-18 as cytokinin deficient loss-of-function G20-18 mutants exhibited impaired biocontrol. Furthermore, biocontrol activity was restored after functional complementation of these strains with cytokinin biosynthetic genes. However, *P*. *fluorescence*–produced cytokinin mediated biocontrol was found to be SA-dependent and it also required active cytokinin perception as shown by Arabidopsis mutant analysis. This shows the potential of such PGPR for incorporation into integrated plant protection programs. Though the SRB02 bacterial strain isolated in this study produce cytokinin in culture, significant reduction in cytokinin levels was observed in SRB02-treated plants grown under both temperature regimes with a concomitant increase in the expression of cytokinin dehydrogenase genes (*GmCKX04* and *GmCKX07*) which degrade cytokinin. Furthermore, antagonistic roles of ABA and cytokinin have been demonstrated during abiotic stresses [116]. Thus, low cytokinin levels in SRB02-treated plants may be due to the negative effects of increase ABA levels in these plants.

Different PGPR such as *Bacillus pumilus* and *B*. *licheniformis* produce gibberellins such as GA1, GA3, GA4, and GA20, and they suppress GA-deficient phenotypes of alder seedlings [32]. Earlier studies also described GA production by different PGPR, such as *Rhizobium phaseoli* [117], *Azospirillum lipoferum* [118], *A*. *brasilense* [119], and others. According to these studies, PGPR cultures contain nanogram amounts of GA1, GA3, GA4, GA9, GA20, and other GA derivatives. PGPR, when applied to plant roots, increase endogenous GA levels in maize [120]. *Promicromonospora* sp. SE188 produced GA1, GA4, GA9, GA12, GA19, GA20, GA24, GA34, and GA53 in culture and significantly enhanced the production of GA12, GA24, GA9, GA4, and GA34 in tomato plants [121]. In this study, we did not observe a direct linear or inverse relationship between the phytohormone profiles of SRB02 and SRB02-treated plant, whether grown under normal temperature or high temperature conditions. For example various GAs measured from SRB02 cultures and SRB02-treated soybean plants did not correlate with each other. Furthermore, various GA derivatives differ in their biological activity, hence further increasing the complexity and significance of PGPR-host plant interaction.

Methyl viologen is a superoxide radical (O˙ˉ_2_) donor. In plants, superoxide radicals and/or other ROS are generated in response to different stresses. These highly reactive and toxic molecules which are mainly produced in the mitochondria and chloroplasts can damage cellular proteins, lipids, carbohydrates, and DNA, resulting in oxidative stress [122]. On the other hand, plants also possess very efficient enzymatic antioxidant defense machinery. Superoxide dismutase (SOD) provides protection against superoxide radicals, while catalase enzymes (CAT) degrade H_2_O_2_. Enzymes such as ascorbate peroxidase (APX), glutathione reductase (GR), monodehydroascorbate reductase (MDHR), glutathione S-transferase (GST), and others, are also important components of plants’ antioxidant machinery. Analysis of SRB02 cultures indicated significantly higher SOD and CAT activities than those observed in AY1 cultures, indicating the presence of highly active and efficient antioxidant machinery in SRB02. Similarly, SRB02 also grew much better on GSNO and CysNO plates. These results parallel the findings of other studies that report oxidative and nitrosative stress tolerant PGPR [19, 123]. However, the question of how PGPR tolerate high oxidative and nitrosative stresses remains largely unanswered. Storz et al. in 1999 reviewed the mechanisms by which several rhizospheric and other bacteria tolerate high oxidative stress [124]. More recently, Shen et al. (2013), through comparative genomic analysis of four PGPR in the genus *Pseudomonas*, showed the presence of ROS-detoxifying enzymes, including 11 peroxidases, 5 catalases, 2 superoxide dismutases, and 19 glutathione S-transferases [123]. Functional components such as the two-component regulator GacS/GacA [125], and SoxR and OxyR [126], are key regulators of oxidative stress tolerance in PGPR. Furthermore, exopolysaccharides and polyhydroxyalkanoates have also been shown to play important roles in oxidative stress tolerance in PGPR [127] and other bacteria [128]. Over-expression of flavodoxin in *E*. *coli*, the nitrogen fixing bacterium *Ensifer meliloti*, and the PGPR *Pseudomonas fluorescens* strain Aur6 enhanced the tolerance of these bacteria to oxidative stress provoked by H_2_O_2_ and paraquat or atrazine; on solid medium which is also in agreement with the findings of this study.

Similarly, microbial enzymes responsible for providing resistance to nitrosative stress have been identified through functional genetics analyses of several non-PGPR bacteria [129–131]. One such enzyme that is highly conserved from bacteria to humans is S-nitrosoglutathione reductase (GSNOR) [131], which controls the global levels of S-nitrosothiols (SNO) through the process of denitrosylation [132]. Other enzymes such as flavorubredoxin nitric oxide reductase and flavohemoglobin denitrosylase play important roles in direct consumption of NO [130, 133], whereas the bacterial AhpC peroxiredoxin proteins are responsible for direct consumption of peroxynitrite. Nitrosative stress tolerance has been studied extensively in bacterial-mammalian host systems, but very little information is available about this phenomenon in PGPR. Information related to the fate of NO in the interactions of plants with rhizospheric bacteria is extremely important as several nitrifying and de-nitrifying bacteria play important roles in nitrogen and NO cycles.

The use of PGPR in agriculture is gaining importance and is becoming a permanent part of cropping systems throughout the world. It is not only environment-friendly but also cost-effective, reliable, and durable. The use of gram-positive PGPR such as the *B*. *aryabhattai* strain SRB02 identified in this study is of special importance as these bacteria have the ability to form resistant endospores. Once applied and established in a field they can survive harsh conditions for a significant period of time. Our results show that *B*. *aryabhattai* strain SRB02 rapidly colonizes the roots of host plants and promotes their growth by regulating the homeostasis of several phytohormones, and it is also highly resistant to oxidative and nitrosative stresses, making it a valuable organism for incorporation into biofertilizers and other soil amendments for the improvement of crop productivity. Further studies are required to determine the molecular mechanisms underpinning oxidative and nitrosative stress tolerance in SRB02 and to exploit this information for engineering PGPR in order to further increase their plant growth promoting abilities.

## Supporting information

S1 Fig Phylogenetic tree analysis of *B*. *aryabhattai* strain SRB02. (TIF)

S2 Fig SEM (Scanning Electron Microscope) images of stomata on leaf surface under high day/night temperatures. (TIF)

S3 Fig Effects of SRB02 on root growth of soybean plant. (TIF)

S1 Table List of primers used for qRT-PCR. (DOCX)
