# Supplementary figures and images for: Bacillus aryabhattai SRB02 tolerates oxidative and nitrosative stress and promotes the growth of soybean by modulating the production of phytohormones
Source: PLoS One. 2017 Mar 10;12(3):e0173203. doi: 10.1371/journal.pone.0173203 (PMC5345817; doi:10.1371/journal.pone.0173203)

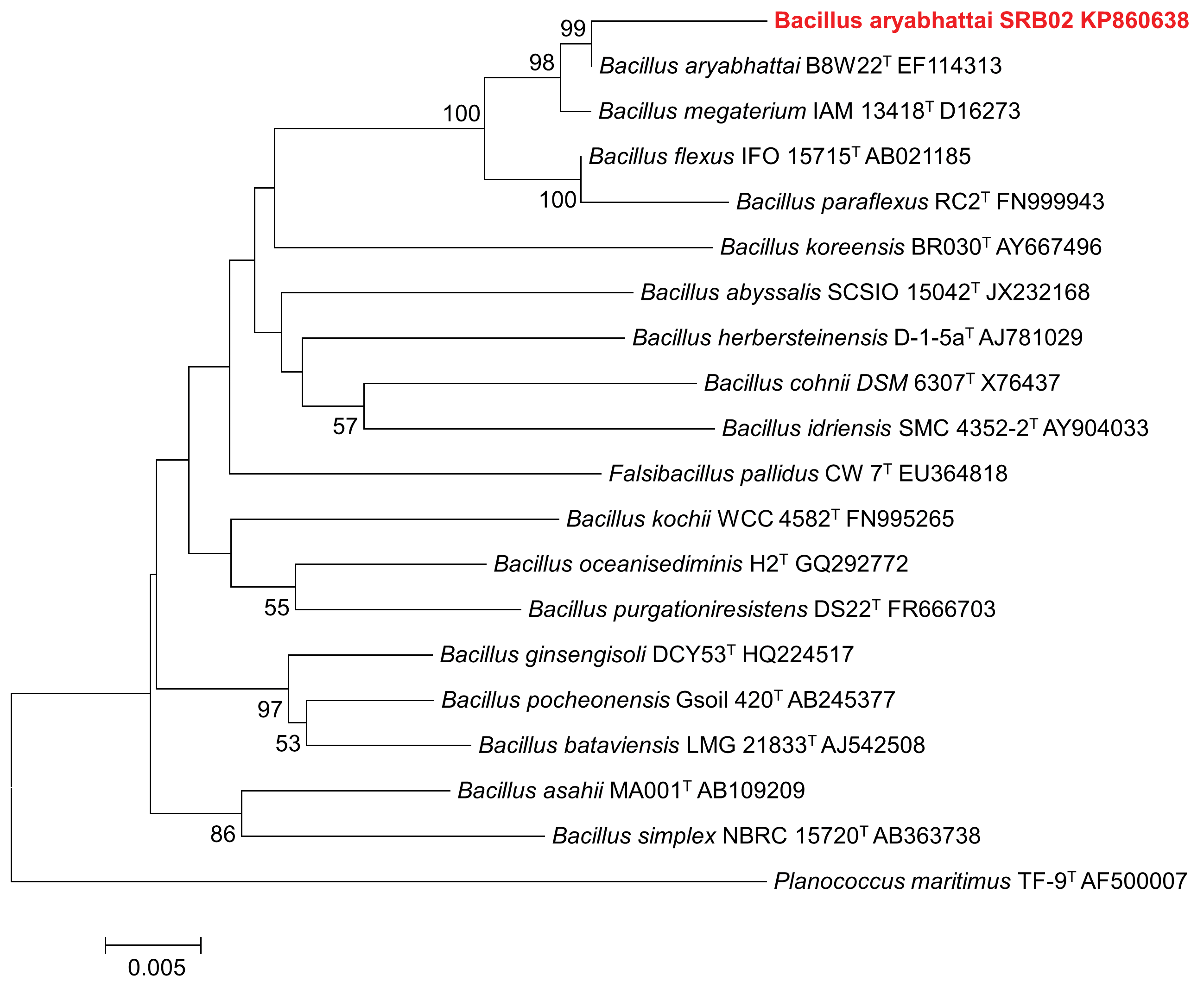

Supplement: S1 Fig — (TIF) [file pone.0173203.s001.tif]

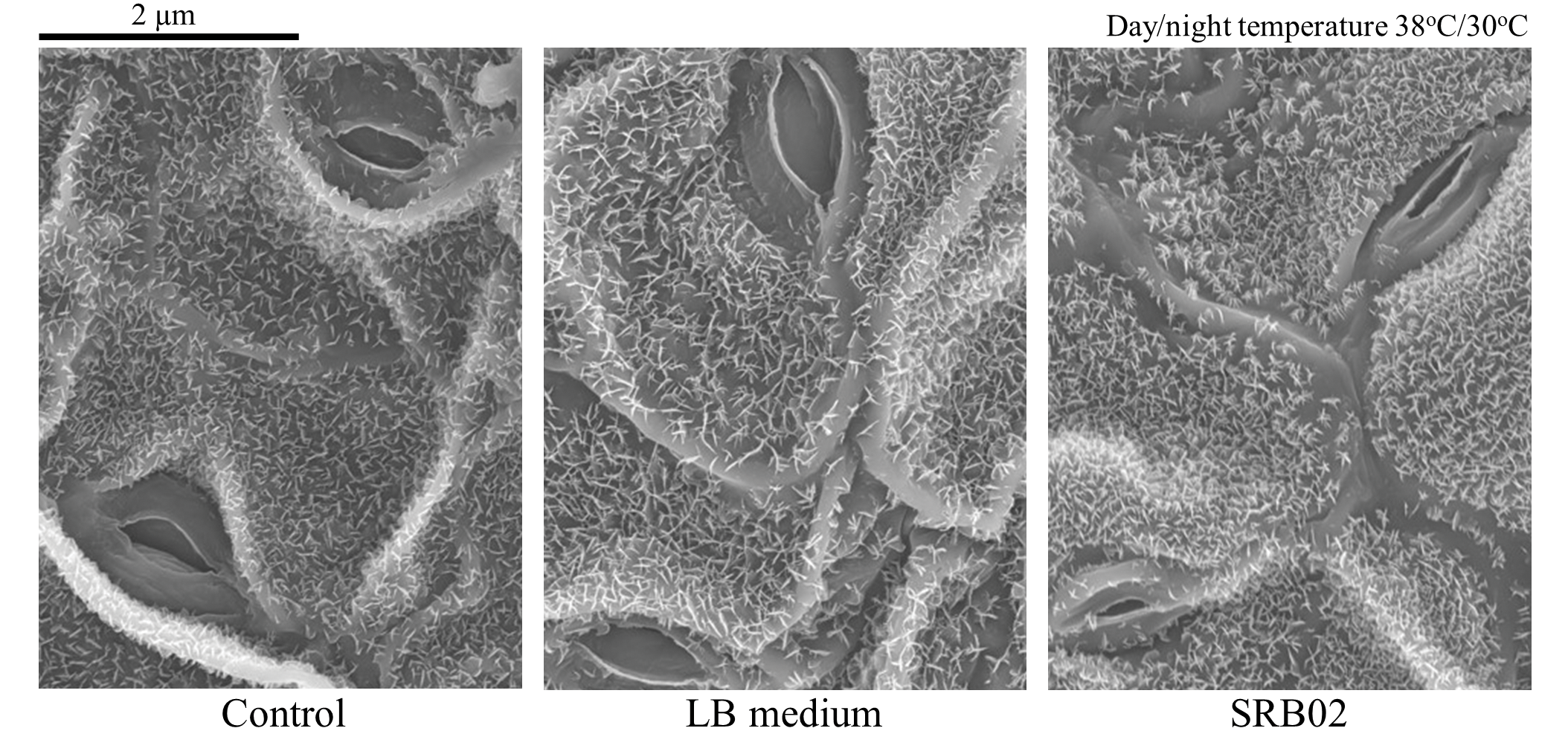

Supplement: S2 Fig — (TIF) [file pone.0173203.s002.tif]

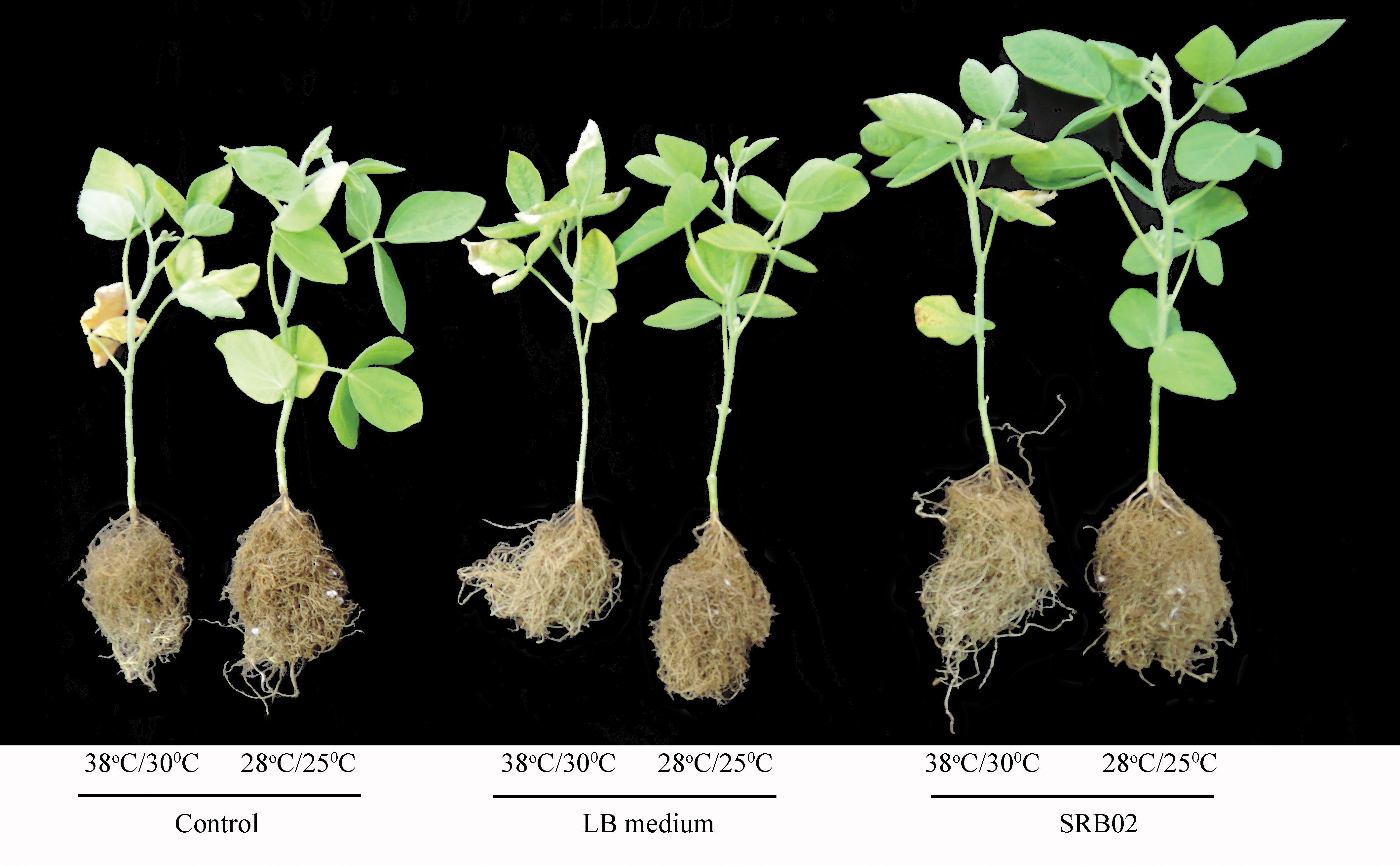

Supplement: S3 Fig — (TIF) [file pone.0173203.s003.tif]
