# Supplementary material for: Bacillus aryabhattai SRB02 tolerates oxidative and nitrosative stress and promotes the growth of soybean by modulating the production of phytohormones
Source: PLoS One. 2017 Mar 10;12(3):e0173203. doi: 10.1371/journal.pone.0173203 (PMC5345817; doi:10.1371/journal.pone.0173203)
Supplement: S1 Table — (DOCX) [file pone.0173203.s004.docx]

**Supplementary Table S1. List of primers used for qRT-PCR**

| **Gene** | **Accession No.** | **Forward primer sequence (5′-3′)** | **Reverse primer sequence (5′-3′)** |
| --- | --- | --- | --- |
| GmZEP3 | Glyma01g39310 | GCCGTTGATATTACTTATCTTGTAAGT | ATCACGCTAGGGCGCTTATGAGTACATA |
| GmNCED (1a+1b) | Glyma05g27250 + Glyma08g10190 | ACCACCTCTTCGACGGCGACGGAATGGT | ATGGCGAGGAGTTTTCCGTTGAAGAAGA |
| GmRD20A | Glyma03g41030 | GTGGCACATGACTGAAGGAA | ATCTTTCCAGCAGCACCTCT |
| GmGA2ox1 | Glyma02g01330 | CTGGTGGCGGAGGGTTT | CACGGGAGGGTATTGATTGA |
| GmGA2ox2 | Glyma10g01380 | CAATCCTTTTGGCTAT | GCTTGCTGAGTGAGAA |
| GmGA3ox1 | Glyma04g07520 | CTGTGCACCCTCACCCTAAT | CGAGCAACAGAGTGAACCAA |
| GmGA3ox2 | Glyma06g07630 | TGCAGGGTTTTGTGATTTGA | CTCGGGACAACTTGGGTAAA |
| GmGA20ox1 | Glyma03g02260 | CCAGCAAAAGTGTGGAGGAT | TGCCAACTCCTAGGGTCATC |
| GmGA20ox2 | Glyma07g08950 | ATGCCAAACCAATCCAACAT | TGCCTCACTCAATTCAGCAC |
| GmIAA16 | Glyma02g16071 | ATGTCCCCGACCTATGAAGACAG | TGCCAGTCCAATAGCTTCCTTTC |
| GmIAA9 | Glyma01g02350 | AGGGGAAGTTCGCTGGTAAT | TGGTTATGACCGGTTCCATT |
| GmaxACT11 | Glyma18g52780 | CGGTGGTTCTATCTTGGCATC | GTCTTTCGCTTCAATAACCCTA |
| GmCKX04 | Glyma09g07360 | TACTTGACGGTGGGAGGAAC | CCAGTGATGACGTCCATTTG |
| GmCKX07 | Glyma15g18560 | ATCTCCACAGTGGGCAAAAC | TTGCAGAAGATGGTGTCGTC |
